# Reduced Wind Speed Improves Plant Growth in a Desert City

**DOI:** 10.1371/journal.pone.0011061

**Published:** 2010-06-10

**Authors:** Christofer Bang, John L. Sabo, Stanley H. Faeth

**Affiliations:** 1 School of Life Sciences, Arizona State University, Tempe, Arizona, United States of America; 2 Faculty of Ecology Evolution and Environmental Sciences, School of Life Sciences, Arizona State University, Tempe, Arizona, United States of America; 3 The University of North Carolina at Greensboro, Greensboro, North Carolina, United States of America; Dalhousie University, Canada

## Abstract

**Background:**

The often dramatic effects of urbanization on community and ecosystem properties, such as primary productivity, abundances, and diversity are now well-established. In most cities local primary productivity increases and this extra energy flows upwards to alter diversity and relative abundances in higher trophic levels. The abiotic mechanisms thought to be responsible for increases in urban productivity are altered temperatures and light regimes, and increased nutrient and water inputs. However, another abiotic factor, wind speed, is also influenced by urbanization and well known for altering primary productivity in agricultural systems. Wind effects on primary productivity have heretofore not been studied in the context of urbanization.

**Methodology/Principal Findings:**

We designed a field experiment to test if increased plant growth often observed in cities is explained by the sheltering effects of built structures. Wind speed was reduced by protecting *Encelia farinosa* (brittlebush) plants in urban, desert remnant and outlying desert localities via windbreaks while controlling for water availability and nutrient content. In all three habitats, we compared *E. farinosa* growth when protected by experimental windbreaks and in the open. *E. farinosa* plants protected against ambient wind in the desert and remnant areas grew faster in terms of biomass and height than exposed plants. As predicted, sheltered plants did not differ from unprotected plants in urban areas where wind speed is already reduced.

**Conclusion/Significance:**

Our results indicate that reductions in wind speed due to built structures in cities contribute to increased plant productivity and thus also to changes in abundances and diversity of higher trophic levels. Our study emphasizes the need to incorporate wind speed in future urban ecological studies, as well as in planning for green space and sustainable cities.

## Introduction

Half of the world's human population lives in cities, and urban ecosystems are the most rapidly expanding ecosystem on the planet [1]. Urbanization trends pose serious problems with respect to ecosystem services and human well-being because the complex ecological processes involved are often underestimated or neglected by urban consumers [2]. Increasing the knowledge of urban effects on ecosystems is fundamental to the understanding of regional and global changes in ecosystem services [3], [4]. Studies of urbanization effects on ecosystem functions and services have become more common, but our working knowledge of these complex systems is still sparse.

Primary productivity and diversity are two ecosystem properties that are directly altered by urbanization (e.g., [5]). These properties provide ecosystem services by maintaining nutrient balance, increasing aesthetic value and creating recreation opportunities for urbanites, and by providing a food base and habitats for urban wildlife. Cities have great potential to achieve high productivity and biodiversity (e.g., [6]), and business developments are increasingly including green areas to support urban biodiversity, with documented benefits to people and wildlife [7], [8], [9]. How to optimize productivity and diversity, however, is far from obvious since there are multiple interacting mechanisms underlying changes in productivity from rural to urban areas, such as nutrient and water supplements and changes in temperature and light regimes [10], [11], [12]. In addition, the non-linear relationship between productivity and species richness (one common measure of biodiversity) is far from clear in all systems, but is considered the dominant model at local scales and across community types [13]. This unimodal relationship suggests productivity is a key factor influencing changes in population density, community structure and species diversity of plants and non-human animals in urban settings [5].

One factor that may affect productivity and hence diversity is reduced wind speed in cities, which is caused by structures such as buildings, walls, embankments, elevated roadways and planted vegetation. Urban areas have significantly lower maximum and average wind speed than do natural areas lacking these structures [14]. Reduced wind speed in urban areas is well documented in cities around the world [15]. For example, Warsaw, Poland, experienced 2 m s^−1^ lower wind speed than the surrounding area throughout the year [14], and the annual mean difference in Berlin, Germany, was found to be 10–20% lower than the surroundings [16]. Other large cities, such as Delhi, India [17], London, UK [18] and Melbourne, Australia [19] also experience low wind speeds. Most of the studies involving wind in cities link reduced wind speed to the urban heat island effect and higher concentrations of pollutants. Studies that consider wind and vegetation typically focus on how vegetation affects the urban wind pattern. Here we examine the reverse chain of cause and effect and ask how wind patterns altered by urbanization affect plant productivity. Changes in urban wind have heretofore not been linked to increases in urban productivity.

Wind is known to affect biotic communities in non-urban systems. For example, protecting agricultural crops from wind increases yields (e.g., [20]). Likewise, wind is a key factor in pollination and seed dispersal (e.g., [21]), affects insect herbivory (e.g., [22]), tree growth [23], and even bat activity (e.g., [24]). Clearly, wind is an important factor on many levels in both natural and urban ecosystems.

Experiments testing the effects of wind on plant growth have been performed since the beginning of the last century using fans, wind tunnels and natural or artificial shelters (e.g., [25], [26], [27], [28]). Yet, there have been no field experiments, to our knowledge, that manipulate wind to determine its effect on plant growth in an urban context. Observations from recent urban ecological field studies suggest that urban plants recover faster after frost events, and that increased plant growth in the city cannot be fully explained by increased water or nutrient availability (C. Bang, unpublished data). Research from New York City suggests that a reduction in ozone concentration in the city core explained increased tree growth [29]. Although ozone is probably important in the desert as well (annually, Phoenix, AZ, has numerous nonattainment days for ozone, especially in the summer [30]), we hypothesized that the reduced wind velocity in the city increases overall plant productivity. We designed an experiment where we compared growth of wind-protected and wind-exposed plants in three habitat types (desert, desert remnants and urban yards), while keeping track of air quality data via local climate monitoring stations. A common native ornamental shrub, *Encelia farinosa* was used in the study. We predicted that in the desert, sheltered plants would grow better than wind-exposed plants, exhibiting increased height, diameter and biomass. Because plants in urban areas were already subjected to reduced wind speeds as a result of surrounding buildings, fences or walls, we expected to see no difference between exposed and sheltered plants in the city. Desert “remnants” are similar in structure to native desert (open space, similar vegetation), but are located within the city. We predicted that sheltered plants in desert remnant habitats would display similar differences in growth to those in the desert, unless some other urban effect such as altered air quality or elevated temperature subsumed the effect of wind.

## Results

### Wind treatment efficacy

**Figure 1:**
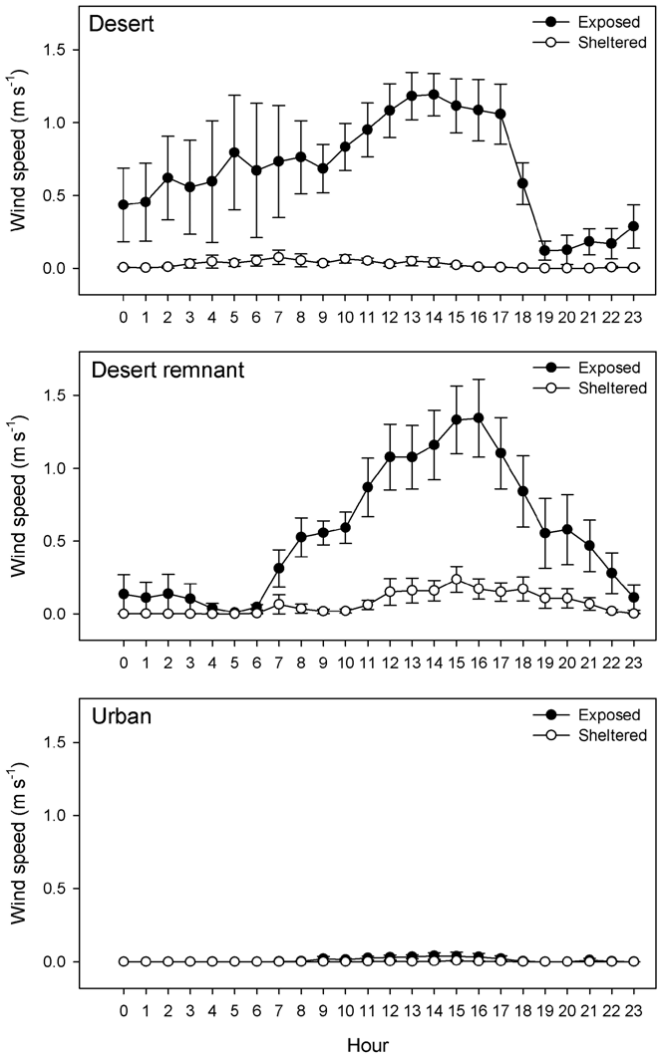
Wind speed in three habitats. Daily average wind speed (m s^−1^) over 10 days in our three habitat types. Error bars are ±1SE.

The windbreak significantly reduced the wind speed in desert and remnant areas similar to levels in the exposed urban habitat (Table 1, Fig. 1). Air temperatures were not altered between sheltered and exposed treatments, except for higher day temperatures in urban areas in the sheltered treatment (Table 1). Soil temperatures were not significantly different in the desert, but urban and remnant had significantly warmer soil in the sheltered treatment. The windbreak did not change relative humidity significantly, but overall the desert had lower humidity than the urban and remnant habitats. Soil moisture was significantly lower in the desert sheltered treatment, but not in the remnant or urban habitats.

**Table 1:** Daily averages of environmental variables measured in the study.

Environmental factor	Habitat	Sheltered	Exposed	Significant difference
Wind speed (m s^−1^)^1^	Desert	0.0266±0.0048	0.6848±0.0511	***
	Remnant	0.0747±0.0113	0.5741±0.0445	***
	Urban	0.0013±0.0004	0.0125±0.0026	***
Air temperature, day (°C)^2^	Desert	27.701±0.520	26.925±0.528	n.s.
	Remnant	28.238±0.512	26.658±0.502	n.s.
	Urban	27.942±0.457	25.285±0.423	***
Air temperature, night (°C)^2^	Desert	19.121±0.466	19.514±0.460	n.s.
	Remnant	18.533±0.470	20.268±0.577	n.s.
	Urban	21.321±0.334	20.782±0.330	n.s.
Soil-temperature (°C)^3^	Desert	24.621±0.368	23.999±0.331	n.s.
	Remnant	24.394±0.387	23.212±0.336	*
	Urban	23.952±0.297	22.449±0.272	**
Relative humidity (%)^4^	Desert	15.109±1.331	14.245±1.348	n.s.
	Remnant	19.256±1.128	17.533±1.102	n.s.
	Urban	17.467±1.008	18.139±1.059	n.s.
Soil moisture (m^3^/m^3^)^3^	Desert	0.1948±0.0077	0.2280±0.0063	*
	Remnant	0.2320±0.0056	0.2326±0.0053	n.s.
	Urban	0.2645±0.0046	0.2579±0.0036	n.s.

Daily averages ±1SE of environmental factors based on hourly averaged data. In cases with unequal variance, we used the Satterthwaite *t*-statistic. Significance is determined using sequential Bonferroni test.

*' **' *** Exposed plants significantly different from sheltered at P<0.05, 0.001 and 0.0001, respectively.

1 n = 10 days.

2 n = 31 days.

3 n = 48 days.

4 n = 33 days.

n.s. = not significant.

### Air quality

Ozone concentrations generally increased from February through May, and increased along a downwind gradient from the southwestern desert area to the northeastern desert area. Concentrations were relatively low in the city core; however, extremes in the hourly measurements (0 to 0.0978 ppm) were both observed in the city. The lowest values were observed at night and the highest during afternoon rush hours in May. Nitrogen oxides (NO_X_) concentrations, on the other hand, generally decreased from February through May, and were highest in the city core. Hourly measurements ranged from 0 to 0.587 ppm. There were no NO_X_ data available for the northeastern desert area. Overall, the city locations experienced greater variance in air quality than outlying localities, with most of the variance attributed to diurnal fluctuations.

### Effects of wind on plant growth

**Figure 2:**
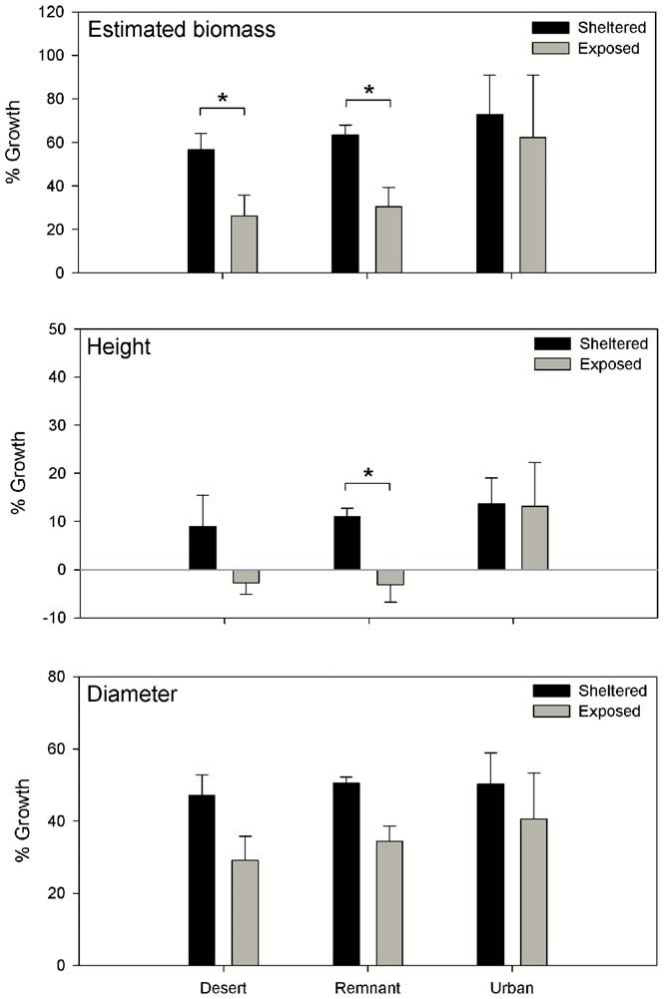
Plant growth. Growth of *E. farinosa* in wind-protected and exposed treatments, February–May 2008, across three habitats with three replicates of each. Percent growth is given for estimated aboveground biomass, height and mean crown diameter. Error bars are standard errors, and asterisks indicate significant pair-wise differences (Tukey-Kramer adjusted 1-tailed P-values).

Assumptions for parametric testing (independence, normality, equal variance) were met for all measured response variables. The treatment effect was significant for all response variables (Type III ANOVA, Table 2). There were no significant effects of habitat or the habitat-treatment interaction. There was no spatial autocorrelation except in two locations, and only one of these was significant for both Moran's I and Geary's C (Table S1). Because the majority of the locations demonstrated no autocorrelation, we ignored any such structure in the variance and assumed that in spite of the clumped design, samples were adequately independent. The change in estimated biomass from February to May 2008 was significantly greater among sheltered plants than exposed plants in desert and remnant areas (Fig. 2). Sheltered desert plants increased in biomass by 56.6±7.6% (mean ± SE) while exposed plants increased 26.3±9.5% (Tukey-Kramer, 1-tailed *P* = 0.00845). Sheltered remnant plants increased in biomass by 63.3±4.8% while exposed plants increased 30.5±8.9% (*P* = 0.00365). There was no significant difference in biomass change between sheltered and exposed plants in urban areas (sheltered 72.8±18.2%, exposed 62.4±28.7%, *P* = 0.4197). Height differences between sheltered and exposed plants were not significant except in the remnant habitat (sheltered 11.0±1.8%, exposed −3.1±3.6%, *P* = 0.0266). The trends, however, are similar to the biomass results (Fig. 2). There were no significant differences in diameter between sheltered and exposed plants.

**Table 2:** Effect of treatments on growth.

Source	DF	Type III SS	Mean Square	F-value	One-tailed
					P-value
*Estimated biomass*					
Habitat	2, 6	24163.58	12081.79	0.92	0.2238
Treatment	1, 166	24373.90	24373.90	21.23	<0.0001
Habitat×Treatment	2, 166	3795.37	1897.69	1.66	0.0971
*Height*					
Habitat	2, 6	5310.21	2655.10	2.25	0.0936
Treatment	1, 166	1960.87	1960.87	9.37	0.0013
Habitat×Treatment	2, 166	861.58	430.79	2.06	0.0652
*Diameter*					
Habitat	2, 6	2024.45	1012.22	0.31	0.3721
Treatment	1, 166	7810.71	7810.71	12.58	0.0003
Habitat×Treatment	2, 166	323.22	161.61	0.26	0.3854

Analysis of variance, mixed model procedure, Type III tests of fixed effects for three growth responses: Estimated biomass, height and average crown diameter. Sum of squares and mean squares are obtained from the generalized linear model procedure, Type III, using site nested in habitat as error term.

## Discussion

The field of urban ecology has recently focused on the ecological services provided by the urban environment, and how we might more wisely manage ecosystems to enhance those services and make cities more livable. One aspect of livable cities is increased green space (e.g., [31]). In cities around the world, plants provide a number of critical ecosystem services, including regulating air quality, water balance, and ground surface temperatures (e.g., [16]). In this paper we provide evidence that the unique urban topography imposed by built structures, such as walls, fences and buildings reduces wind speeds and increases growth and biomass accumulation of a common native ornamental plant. This finding has broader implications for the services urban ecosystems provide. For example, increased productivity due to reduced wind speed may partially offset CO_2_ emissions in cities. Moreover, since higher plant productivity may be correlated with increased species richness (e.g., [32]), reduced wind speeds in cities may increase species richness of animals that can benefit from faster growing plants.

### Plant growth

Our results for *E. farinosa* in the metropolitan Phoenix area strongly suggest that reduced wind speed improves plant growth. We have demonstrated that plants protected against wind in natural environments, such as the Sonoran Desert, increased twofold in biomass compared to unprotected plants. Consistent with our predictions, the sheltered plants in both desert and desert remnant habitats responded similarly to plants growing in urban areas. Also consistent with our predictions, extra wind protection in urban habitats did not have any effect on plant growth, because plants there are already sheltered by the structural design of the city.

Reduced wind speed in urban habitats enhances aboveground growth, but it is difficult to pinpoint the underlying physiological mechanisms. Wind speed determines leaf boundary layer conductance, which directly influences photosynthetic rate, transpiration rate, and leaf temperature via the energy balance equation [33]. In addition, the boundary layer alters variables influencing guard cells and can indirectly control stomatal conductance [34]. Although our study was not designed to test the exact mechanism, we believe that the combination of high water availability and reduced wind speed allow stomata to remain open, contributing to the increased gas exchange observed by Martin and Stabler [35]. Reduced wind speed also reduces chances for mechanical damage [28] and thigmomorphogenetic responses (altered growth as response to mechanical stimulation [36]). Lack of mechanical stimulation can lead to stem elongation and poorly developed root systems [37]. We did however not measure root extension.

### Desert remnants and urban ecology

The effects of wind speed on primary productivity are likely highly variable within the city, given that wind speeds vary with proximity to built structures [38]. Our desert remnant sites are examples of localities within the city that experience wind speeds comparable to the outlying desert (albeit somewhat lower, Fig. 2, Table 1), and consequently have reduced plant growth similar to the desert sites.

Remnant areas provide urban ecologists with a unique form of experimental control. In our setup, desert remnants serve as “true replicas” of desert located within the city development, and are thereby generally exposed to the same heat island effect and the fluctuating concentrations of pollutants (e.g., NO_X_, ozone) and CO_2_ as other urban localities. Ozone and NO_X_ concentrations varied from one side of the city to the other, without having any visible effect on the plants growing in the desert locations at each side of the city. If air quality factors are important in controlling plant growth in this city as observed in other cities [29], the plant growth in the remnant areas would be more similar to the urban plant growth than to outlying desert plant growth. Instead, remnants show the same growth patterns as the desert when either sheltered or unsheltered, despite all the other factors experienced by urban plants. This suggests, at least in this desert city, that wind is a key factor for plant growth, and may override more well-studied factors, such as altered temperatures and air quality.

Because our study was of a relatively short-term character (four months), and the plants were in pots, we cannot exclude legacy effects in soil [39] or the potential long-term effects of changes in air quality [29], nutrient depositions (Hall et al. *in review*) or air temperature [40]. Whereas altered wind patterns due to built structures are common in all cities [38], topography and vegetation surrounding the city determine the magnitude of wind speed differences. For example, Seoul, South Korea, is surrounded by forest and agricultural land, and the wind speed is in fact higher in the city than the rural areas [41]. Studies from Beijing, China, demonstrate diurnal, seasonal and spatial variation in wind speed depending on topographical factors such as building design, road corridors and surrounding landscape [42], [43]. This complex relationship is also found elsewhere. For example in Buenos Aires, Argentina, a change in wind direction can lead to an inverse heat island effect [44]. All of these factors likely play a role in primary production and diversity in cities, and thus require further experimental studies [45].

### Carbon sequestration and biodiversity

Cities are major sources of CO_2_ and are thus large contributors to the global increase in atmospheric CO_2_ [46]. Although the magnitude of carbon storage by urban trees is relatively small compared to emissions from burning of fossil fuel [47] urban forests may provide an important ecosystem service in terms of carbon balance [48]. Martin and Stabler [35] estimated that plants in urban residential yards acquired 2.8 times more atmospheric carbon than plants in desert sites. Because our study suggests that wind is an important driver of plant growth and productivity in cities, understanding the effect of wind will be important in urban design and landscaping to optimize carbon storage.

Because cities are the most rapidly expanding habitat worldwide, urban planners and conservation biologists are increasingly interested in the contribution of cities to diversity [49], rather than dismissing them as habitats where diversity often declines [50]. Primary productivity is often linked with higher species richness of both plants and higher trophic levels, albeit in a unimodal pattern (e.g., [32]). Changes in plant productivity may therefore cascade upward to alter trophic dynamics in arthropod and bird communities [51]. Future studies in urban ecology could focus on higher trophic levels along a productivity gradient, to which we have provided a simple way to manipulate productivity. As new efforts in urban landscape design seek to increase the amount and heterogeneity of green spaces to maintain or enhance biodiversity in cities (e.g., [52]), it will be imperative to consider altered wind patterns in cities and their effects on plant growth and productivity.

## Materials and Methods

### Study plant

We chose the native shrub brittlebush (*Encelia farinosa* Gray ex Torr. [Asteraceae]) because of its ubiquity in the Sonoran Desert, and because, as a popular landscaping plant in the Phoenix metropolitan area, it occurs in all habitats studied. The physiological characteristics of *E. farinosa* related to photosynthetic optima, carbon assimilation, drought adaptation, heat tolerance, and seasonal morphological changes have been extensively described (e.g., [53], [54], [55], [56]). Others have described variation among and within populations (e.g., [57], [58], [59]), and chemical defense properties (e.g., [60]). In brief, *E. farinosa* respond to seasonal water stress at the end of rainy seasons by replacing larger leaves with smaller, pubescent leaves. Small pubescent leaves reduce water loss (lower surface area and fewer stomata) and maintain lower leaf temperatures due to reflection of radiation by leaf hairs. Smaller leaves also reduce the total photosynthetic capacity of the plant. As summer temperatures rise and water becomes scarce, *E. farinosa* eventually drop all their leaves and remain dormant until the next rainy season, when they quickly respond to the available soil water through rapid CO_2_ uptake, leaf production, and stem growth [56]. Differences in water availability are reflected in size differences between plants in wet and dry areas; the size of *E. farinosa* is documented to increase by 35% in irrigated versus non-irrigated gardens [61]. Despite abundant information on physiological responses of this plant, no studies have directly considered the effects of wind on *E. farinosa* growth.

### Study sites and design

The metropolitan area of Phoenix, Arizona, is situated in the northern end of the Sonoran Desert. The metropolitan area is a widespread heterogeneous patchwork of impervious surfaces and human made landscapes, interspersed with remnants of the Sonoran desert, pasture and irrigated cropland [62]. The majority of residential houses are single family one story homes with large garages, or two-story apartment complexes [63]. High-rise buildings are generally restricted to downtown areas in Phoenix, and are not typical for this city. Our study area ranged from approximately 300 m above sea level (asl) southwest of the city to 600 m asl east of the city. Most of the urban locations were at 350 m asl. To compare urbanized habitats with natural habitats, we identified three habitat categories: outlying desert, desert remnants, and urban sites; and we selected three replicate sites for each habitat type. Outlying desert sites were typical of the Sonoran Desert with scattered perennials such as creosote bush (*Larrea tridentata*), bursage (*Ambrosia deltoidea*), cholla cacti (*Cylindropuntia* spp.), palo verde trees (*Parkinsonia* spp.), ironwood trees (*Olneya tesota*) and other *E. farinosa*. Sites were generally flat with open soil in the spaces between the shrubs or trees. Desert remnants were defined as natural desert patches of varying sizes that have become islands in the urban landscape, completely surrounded by, or at the fringe of, urban development. They were similar to the desert sites in structure and vegetation, but we assumed that remnants had similar air quality conditions as nearby urban sites and experienced general climatic changes associated with the city (e.g., urban heat island). Since urban locations could potentially be very different in terms of wind speed, we chose sites near different building structures. One of the locations was inside an open garden on Arizona State University, Tempe campus, surrounded by buildings approximately 15 m tall. The second urban location was also on Tempe campus, but located in a potential wind corridor (between two buildings, 14 and 15.2 m tall). The third urban location was an empty lot adjacent to one major highway, sheltered by a 1.5 m fence and one 3 m tall mesquite tree. The urban locations represent typical urban commercial settings of the region.

**Figure 3:**
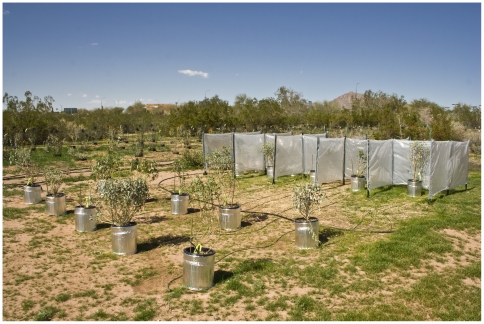
Experimental setup. The windbreak at one of the desert remnant locations. Exposed plants in the foreground and sheltered plants in the back. The plants were in insulated 5-gallon pots (≈18.9 L) with individual drip irrigation ensuring optimal water availability. (Photo: CB).

At each site, we placed 20 potted *E. farinosa* which were obtained from local nurseries (seeds collected locally, all plants approximately seven months old and 67.2±11.2 cm tall, average ±1 standard deviation). Ten plants were randomly assigned for protection by a windbreak shield, while the other ten plants had similar alignment, but without the windbreak (Fig. 3). Wind direction was determined prior to position of the windbreak to ensure optimal functioning [64], since the winds in the Phoenix area tend to be diurnal – upslope in daytime, and downslope at night [30]. Because slope (north or south facing) may affect plant growth by altering soil-temperature and moisture [65], all of our plants were placed on flat ground.

We chose a windbreak design so as to limit any impacts of shading and to allow us to directly measure the effect of wind. Some wind turbulence is inevitable when constructing wind barriers [66], but occasional accelerated wind speed at ground level is a common feature of the urban climate [38]. Our low-cost solution consisted of 18 fence posts (1.52 m long) arranged in a grid, with poultry netting stretched between them creating 10 cubicles of approximately 1.2 m×1.2 m×1.2 m each. Clear plastic sheets (0.1524 mm thick) were sewn to the poultry netting creating the wind barrier. The sheet reduced direct sunlight by 18% during some parts of the day; however light is likely at saturating levels in this region [67]. Air was able to circulate freely because one side was left open, there was no roof, and a 15–20 cm opening was left near the ground, thereby preventing any greenhouse effects. Grass and annuals growing around the pots and windbreak were regularly removed to improve air circulation.

All plants received ample watering (2 L drip twice per day), and were grown in insulated pots (to moderate root temperatures of all plants since they were above ground) to exclude confounding effects of different soil types in the desert versus the urban area. The pots contained soil consisting of ¼ native top soil and ¾ composted mulch, and two tablespoons of Osmocote® slow-release fertilizer to maintain a sufficient soil nutrient level. Since our biomass estimates did not include reproductive parts (Supporting Information S1, Fig. S1), flower buds were cut off regularly to ensure maximal allocation to vegetative growth [68], [69], [70]. Wind speed, air temperature and relative humidity (% RH) were measured 0.8 m above ground (at plant level), and soil moisture and soil-temperature were measured 5–15 cm below the soil surface in the middle of the pot. We used OWL2pe data loggers with soil-temperature probes (EME Systems) and Davis Instruments cup-anemometers for wind speed, and HOBO® Micro Stations (Onset Computer Corporation) for air temperature, % RH, soil moisture and additional soil-temperature measures. Equipment malfunction and rodents chewing on cables kept us from obtaining continuous climate data throughout the growing season, but the reported time periods are nonetheless representative. Local air quality data were obtained from Maricopa County Air Quality Department (MCAQD) and the Arizona Department of Environmental Quality (ADEQ) networks. This provided quantitative and qualitative information about major local differences in ozone and NO_X_ concentrations between the city core and outlying desert areas.

Plant growth was measured monthly from February to May 2008, and final growth reported in terms of estimated biomass, height and crown diameter [71]. Biomass was estimated based on an equation developed by measuring and weighing the dry mass of *E. farinosa* plants (Supporting Information S1, Figs. S1 and S2). Since there may be discrepancies between stem elongation and actual biomass allocation [37], we also performed analyses on height and diameter.

### Statistical analyses

Statistical tests were performed using SAS® (Version 9.2 for Windows, SAS Institute, Inc., Cary NC, USA). Environmental factors were measured in one of each habitat category and compared (exposed vs. sheltered) with two-sample t-tests using the PROC TTEST procedure. In cases with unequal variance, we used the Satterthwaite *t*-statistic. We used sequential Bonferroni correction for the significance tests [72]. To allow for a general interpretation about habitat, we treated sites as nested within habitat. All response variable data were tested for normality using normal probability plots, and homogeneity of variance was evaluated by plotting residuals versus predicted values from a preliminary fixed factor model. A mixed model with habitat, treatment and the interaction term was analyzed using PROC MIXED and PROC GLM in SAS. Extensive earlier ecophysiological work describing negative effects of wind speed on plant growth in general (e.g., [73], [74], [27], [75], [23]), justified the *a priori* hypothesis that wind-protected plants would deviate positively from wind-exposed plants, in terms of biomass, height and diameter. We therefore report one-tailed *P*-values for the post-hoc comparisons. Type III sums of squares were evaluated and multiple comparisons were based on Tukey-Kramer adjusted *P*-values. To ensure that there was no cross-contamination of the windbreak effect on the exposed treatment plants, the pots within treatments were clumped together (Fig. 1). This compromise made the experiment vulnerable to potential non-demonic intrusions (*sensu* [76]). To see if placement had any effect on plant growth regardless of treatment, we tested for spatial autocorrelation using PASSaGE 2 (beta version, used with permission, Supporting Information S1). Moran's I (global spatial autocorrelation) and Geary's C (local spatial autocorrelation) for each site is listed in Table S1.

## Supporting Information

Supporting Information S1 Description of method used for biomass estimation, and test for spatial autocorrelation.
(0.04 MB DOC)

Figure S1 Schematic drawing of an *E. farinosa* in a 5-gallon (≈18.9 L) pot, side view (left) and top view (right). The letters indicate the monthly measures to estimate aboveground drymass.
(0.60 MB TIF)

Figure S2 Relationship between the height×diameter and aboveground drymass of brittlebush, *E. farinosa* (R^2^ = 0.8223, n = 360). The dotted lines indicate a 95% confidence interval.
(0.07 MB TIF)

Table S1 Test for spatial autocorrelation.
(0.20 MB DOC)
