# Supplementary material for: Reduced Wind Speed Improves Plant Growth in a Desert City
Source: PLoS One. 2010 Jun 10;5(6):e11061. doi: 10.1371/journal.pone.0011061 (PMC2883576; doi:10.1371/journal.pone.0011061)
Supplement: Supporting Information S1 — Description of method used for biomass estimation, and test for spatial autocorrelation. (0.04 MB DOC) [file pone.0011061.s001.doc]

**SUPPORTING INFORMATION**

**for**

**“Reduced wind speed improves plant growth in a desert city”**

**C. Bang, J. L. Sabo and S. H. Faeth**

***Aboveground biomass estimation***

In spring 2006, after measuring height and four diameters of 360 *Encelia farinosa* plants at different sizes grown in 5-gallon (≈ 18.9 L) pots (Fig. S1, modified from [1]), we cut them off near the soil surface, shredded them with a garden shredder and stored them in separate paperbags on the roof of one of the buildings at Arizona State University, Tempe Campus. Temperatures in May-June were generally above 30 °C and the plant material were sun exposed daily. When the bags ceased loosing weight, the drymass was measured for each plant using a precision scale. These measures were then compared with the height and diameter measures and the following relationship were established using simple linear regression using R version 2.6.1 [2] (Fig. S2):

ln(B) = 1.01691 × ln(H × D) - 2.94134 (Eq. 1)

where B is dry biomass (g), H = height (cm) and D is the average of four crown diameters (d_1_-d_4_, Fig. S1). A leaning plant would be measured as relatively short, but with one of the diameters considerably longer than the other three.

***Test for spatial autocorrelation***

Although spatial autocorrelation is generally not recommended for fewer than 30 localities [3], we used the software PASSaGE 2 (http://www.passagesoftware.net, beta version, used with permission) to test if the placement of plants could bias our results. We assumed randomly distributed data, and we optimized lag distance to contain a minimum of 30 pairs per class [4]. We used inverse distance weighting and 1000 permutations. The last class was excluded if < 30 pairs. All *P*-values are Bonferroni corrected (Table S1).

***References***

1. Murray RB, Jacobson MQ (1982) An evaluation of dimension analysis for predicting shrub biomass. Journal of Range Management 35: 451-454.

2. R Development Core Team (2007) R: A language and environment for statistical computing. 2.6.1 ed. Vienna, Austria.

3. Legendre P, Fortin MJ (1989) Spatial pattern and ecological analysis. Vegetatio 80: 107-138.

4. Journel AG, Huijbregts CJ (1978) Structural analysis. Mining geostatistics. New York: Academic Press. pp. 148-236.
