# Supplementary figures and images for: Reduced Wind Speed Improves Plant Growth in a Desert City
Source: PLoS One. 2010 Jun 10;5(6):e11061. doi: 10.1371/journal.pone.0011061 (PMC2883576; doi:10.1371/journal.pone.0011061)

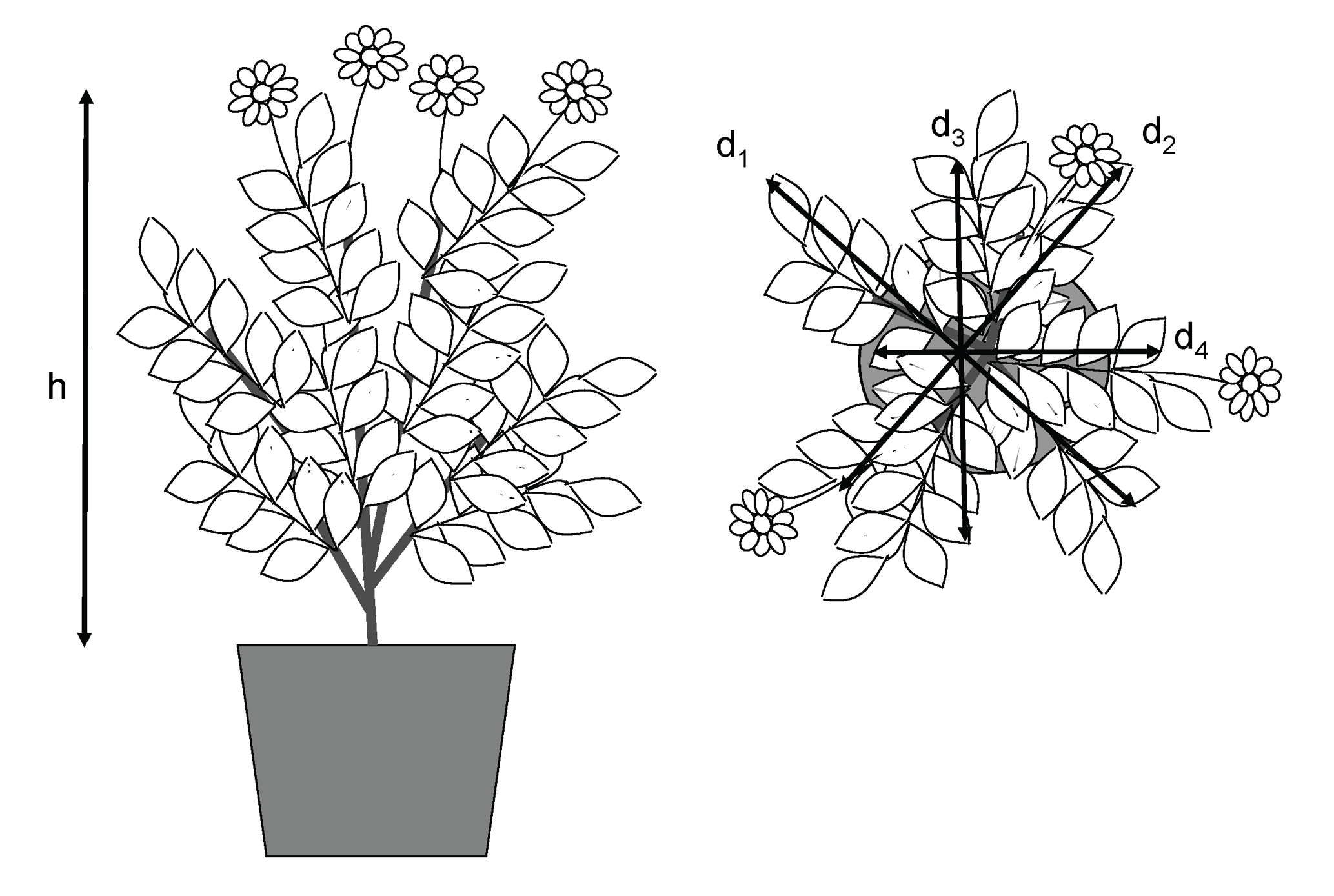

Supplement: Figure S1 — Schematic drawing of an E. farinosa in a 5-gallon (≈18.9 L) pot, side view (left) and top view (right). The letters indicate the monthly measures to estimate aboveground drymass. (0.60 MB TIF) [file pone.0011061.s002.tif]
