# Supplementary material for: Reduced Wind Speed Improves Plant Growth in a Desert City
Source: PLoS One. 2010 Jun 10;5(6):e11061. doi: 10.1371/journal.pone.0011061 (PMC2883576; doi:10.1371/journal.pone.0011061)
Supplement: Table S1 — Test for spatial autocorrelation. (0.20 MB DOC) [file pone.0011061.s004.doc]

**Table S1:** Test for spatial autocorrelation in nine sites. N = 20 at each site.

**Estrella Mtn. Regional Park (Desert)**

| Distance classes | | |  | Moran’s I | | |  |  |  | Geary’s C | | |  |  |  |
| --- | --- | --- | --- | --- | --- | --- | --- | --- | --- | --- | --- | --- | --- | --- | --- |
| # | Min | Max | Pairs | E(I) | I | SD(I) | Z-score | Prob | RandProb | E(c) | c | SD(c) | Z-score | Prob | RandProb |
| 1 | 0 | 1.697 | 31 | -0.053 | -0.056 | 0.162 | 0.020 | 0.984 | 0.774 | 1.000 | 0.861 | 0.178 | 0.781 | 0.435 | 0.463 |
| 2 | 1.697 | 2.720 | 32 | -0.053 | -0.228 | 0.160 | 1.092 | 0.275 | 0.177 | 1.000 | 1.150 | 0.189 | 0.791 | 0.429 | 0.433 |
| 3 | 2.720 | 4.800 | 30 | -0.053 | -0.081 | 0.157 | 0.179 | 0.858 | 0.595 | 1.000 | 1.411 | 0.256 | 1.607 | 0.108 | 0.100 |
| 4 | 4.800 | 6.207 | 34 | -0.053 | 0.054 | 0.142 | 0.752 | 0.452 | 0.692 | 1.000 | 1.304 | 0.250 | 1.217 | 0.224 | 0.247 |
| 5 | 6.207 | 7.692 | 32 | -0.053 | 0.067 | 0.148 | 0.806 | 0.421 | 0.623 | 1.000 | 0.695 | 0.255 | 1.199 | 0.231 | 0.255 |
| 6 | 7.692 | 11.335 | 31 | -0.053 | -0.110 | 0.140 | 0.408 | 0.683 | 0.375 | 1.000 | 0.672 | 0.320 | 1.025 | 0.306 | 0.333 |

Moran's I correlogram P = 1.00000, Geary's c correlogram P = 0.64881

**Usery Mtn. Regional Park (Desert)**

| Distance classes | | |  | Moran’s I | | |  |  |  | Geary’s C | | |  |  |  |
| --- | --- | --- | --- | --- | --- | --- | --- | --- | --- | --- | --- | --- | --- | --- | --- |
| # | Min | Max | Pairs | E(I) | I | SD(I) | Z-score | Prob | RandProb | E(c) | c | SD(c) | Z-score | Prob | RandProb |
| 1 | 0 | 2.140 | 33 | -0.056 | 0.419 | 0.160 | 2.962 | 0.003 | 0.012 | 1.000 | 0.474 | 0.167 | 3.155 | 0.002 | 0.002 |
| 2 | 2.140 | 3.394 | 32 | -0.056 | 0.315 | 0.164 | 2.263 | 0.024 | 0.054 | 1.000 | 0.630 | 0.165 | 2.245 | 0.025 | 0.034 |
| 3 | 3.394 | 4.410 | 31 | -0.056 | -0.105 | 0.158 | 0.310 | 0.757 | 0.517 | 1.000 | 0.856 | 0.188 | 0.763 | 0.445 | 0.424 |
| 4 | 4.410 | 5.463 | 32 | -0.056 | -0.278 | 0.165 | 1.344 | 0.179 | 0.100 | 1.000 | 1.210 | 0.155 | 1.354 | 0.176 | 0.224 |
| 5 | 5.463 | 7.658 | 37 | -0.056 | -0.224 | 0.145 | 1.160 | 0.246 | 0.167 | 1.000 | 1.237 | 0.159 | 1.492 | 0.136 | 0.221 |

Moran's I correlogram P = 0.01530, Geary's c correlogram P = 0.00803

**White Tank Mtn. Regional Park (Desert)**

| Distance classes | | |  | Moran’s I | | |  |  |  | Geary’s C | | |  |  |  |
| --- | --- | --- | --- | --- | --- | --- | --- | --- | --- | --- | --- | --- | --- | --- | --- |
| # | Min | Max | Pairs | E(I) | I | SD(I) | Z-score | Prob | RandProb | E(c) | c | SD(c) | Z-score | Prob | RandProb |
| 1 | 0 | 1.697 | 30 | -0.053 | 0.050 | 0.167 | 0.612 | 0.540 | 0.773 | 1.000 | 0.796 | 0.175 | 1.166 | 0.244 | 0.253 |
| 2 | 1.697 | 2.683 | 41 | -0.053 | 0.099 | 0.137 | 1.101 | 0.271 | 0.505 | 1.000 | 0.806 | 0.173 | 1.122 | 0.262 | 0.267 |
| 3 | 2.683 | 3.795 | 36 | -0.053 | -0.071 | 0.146 | 0.126 | 0.900 | 0.656 | 1.000 | 1.183 | 0.186 | 0.984 | 0.325 | 0.341 |
| 4 | 3.795 | 4.948 | 31 | -0.053 | -0.077 | 0.160 | 0.152 | 0.879 | 0.637 | 1.000 | 1.286 | 0.199 | 1.440 | 0.150 | 0.169 |
| 5 | 4.948 | 6.788 | 39 | -0.053 | -0.197 | 0.138 | 1.050 | 0.294 | 0.166 | 1.000 | 0.999 | 0.182 | 0.003 | 0.998 | 0.995 |

Moran's I correlogram P = 1.00000, Geary's c correlogram P = 0.74971

**ASU Community Services Bldg. (Remnant)**

| Distance classes | | |  | Moran’s I | | |  |  |  | Geary’s C | | |  |  |  |
| --- | --- | --- | --- | --- | --- | --- | --- | --- | --- | --- | --- | --- | --- | --- | --- |
| # | Min | Max | Pairs | E(I) | I | SD(I) | Z-score | Prob | RandProb | E(c) | c | SD(c) | Z-score | Prob | RandProb |
| 1 | 0 | 1.697 | 30 | -0.053 | -0.070 | 0.152 | 0.115 | 0.909 | 0.682 | 1.000 | 1.204 | 0.197 | 1.032 | 0.302 | 0.300 |
| 2 | 1.697 | 2.864 | 41 | -0.053 | 0.003 | 0.128 | 0.432 | 0.666 | 0.986 | 1.000 | 1.068 | 0.162 | 0.416 | 0.678 | 0.684 |
| 3 | 2.864 | 3.800 | 30 | -0.053 | -0.029 | 0.147 | 0.161 | 0.872 | 0.844 | 1.000 | 1.032 | 0.313 | 0.103 | 0.918 | 0.928 |
| 4 | 3.800 | 4.800 | 42 | -0.053 | -0.095 | 0.123 | 0.346 | 0.729 | 0.505 | 1.000 | 1.114 | 0.147 | 0.777 | 0.437 | 0.452 |
| 5 | 4.800 | 5.546 | 31 | -0.053 | -0.029 | 0.143 | 0.168 | 0.867 | 0.823 | 1.000 | 0.638 | 0.312 | 1.162 | 0.245 | 0.270 |

Moran's I correlogram P = 1.00000, Geary's c correlogram P = 1.00000

**Desert Botanical Garden (Remnant)**

| Distance classes | | |  | Moran’s I | | |  |  |  | Geary’s C | | |  |  |  |
| --- | --- | --- | --- | --- | --- | --- | --- | --- | --- | --- | --- | --- | --- | --- | --- |
| # | Min | Max | Pairs | E(I) | I | SD(I) | Z-score | Prob | RandProb | E(c) | c | SD(c) | Z-score | Prob | RandProb |
| 1 | 0 | 1.697 | 30 | -0.053 | 0.139 | 0.165 | 1.159 | 0.247 | 0.440 | 1.000 | 0.862 | 0.176 | 0.784 | 0.433 | 0.432 |
| 2 | 1.697 | 3.000 | 36 | -0.053 | 0.169 | 0.149 | 1.486 | 0.137 | 0.302 | 1.000 | 0.924 | 0.179 | 0.422 | 0.673 | 0.705 |
| 3 | 3.000 | 4.200 | 35 | -0.053 | -0.188 | 0.140 | 0.962 | 0.336 | 0.204 | 1.000 | 1.104 | 0.245 | 0.426 | 0.670 | 0.654 |
| 4 | 4.200 | 4.837 | 34 | -0.053 | -0.323 | 0.154 | 1.759 | 0.079 | 0.049 | 1.000 | 1.208 | 0.153 | 1.357 | 0.175 | 0.197 |
| 5 | 4.837 | 5.909 | 39 | -0.053 | -0.001 | 0.138 | 0.370 | 0.711 | 0.996 | 1.000 | 0.878 | 0.172 | 0.708 | 0.479 | 0.468 |

Moran's I correlogram P = 0.39282, Geary's c correlogram P = 0.87462

**South Mountain Park (Remnant)**

| Distance classes | | |  | Moran’s I | | |  |  |  | Geary’s C | | |  |  |  |
| --- | --- | --- | --- | --- | --- | --- | --- | --- | --- | --- | --- | --- | --- | --- | --- |
| # | Min | Max | Pairs | E(I) | I | SD(I) | Z-score | Prob | RandProb | E(c) | c | SD(c) | Z-score | Prob | RandProb |
| 1 | 0 | 1.700 | 31 | -0.053 | 0.229 | 0.161 | 1.749 | 0.080 | 0.192 | 1.000 | 0.813 | 0.204 | 0.917 | 0.35919 | 0.381 |
| 2 | 1.700 | 3.225 | 32 | -0.053 | 0.202 | 0.157 | 1.615 | 0.106 | 0.234 | 1.000 | 1.004 | 0.214 | 0.016 | 0.98685 | 0.990 |
| 3 | 3.225 | 4.427 | 32 | -0.053 | -0.068 | 0.151 | 0.100 | 0.920 | 0.646 | 1.000 | 0.897 | 0.234 | 0.439 | 0.66083 | 0.673 |
| 4 | 4.427 | 5.664 | 32 | -0.053 | -0.436 | 0.153 | 2.499 | 0.012 | 0.014 | 1.000 | 1.570 | 0.218 | 2.618 | 0.00884 | 0.007 |
| 5 | 5.664 | 7.185 | 32 | -0.053 | -0.186 | 0.147 | 0.903 | 0.366 | 0.197 | 1.000 | 0.906 | 0.254 | 0.372 | 0.70987 | 0.717 |

Moran's I correlogram P = 0.06227, Geary's c correlogram P = 0.04422

**ASU Surplus Facility (Urban)**

| Distance classes | | |  | Moran’s I | | |  |  |  | Geary’s C | | |  |  |  |
| --- | --- | --- | --- | --- | --- | --- | --- | --- | --- | --- | --- | --- | --- | --- | --- |
| # | Min | Max | Pairs | E(I) | I | SD(I) | Z-score | Prob | RandProb | E(c) | c | SD(c) | Z-score | Prob | RandProb |
| 1 | 0 | 1.697 | 34 | -0.053 | 0.059 | 0.156 | 0.716 | 0.474 | 0.730 | 1.000 | 0.853 | 0.170 | 0.864 | 0.388 | 0.360 |
| 2 | 1.697 | 3.046 | 34 | -0.053 | 0.232 | 0.155 | 1.840 | 0.066 | 0.148 | 1.000 | 0.780 | 0.192 | 1.148 | 0.251 | 0.233 |
| 3 | 3.046 | 4.665 | 33 | -0.053 | 0.019 | 0.146 | 0.491 | 0.623 | 0.886 | 1.000 | 0.840 | 0.242 | 0.661 | 0.509 | 0.477 |
| 4 | 4.665 | 6.400 | 37 | -0.053 | -0.213 | 0.140 | 1.147 | 0.251 | 0.131 | 1.000 | 1.132 | 0.203 | 0.652 | 0.514 | 0.523 |
| 5 | 6.400 | 8.410 | 36 | -0.053 | -0.318 | 0.137 | 1.939 | 0.052 | 0.040 | 1.000 | 1.358 | 0.233 | 1.537 | 0.124 | 0.128 |

Moran's I correlogram P = 0.26221, Geary's c correlogram P = 0.62170

**ASU Compound Garden (Urban)**

| Distance classes | | |  | Moran’s I | | |  |  |  | Geary’s C | | |  |  |  |
| --- | --- | --- | --- | --- | --- | --- | --- | --- | --- | --- | --- | --- | --- | --- | --- |
| # | Min | Max | Pairs | E(I) | I | SD(I) | Z-score | Prob | RandProb | E(c) | c | SD(c) | Z-score | Prob | RandProb |
| 1 | 0 | 1.697 | 30 | -0.053 | -0.264 | 0.169 | 1.251 | 0.211 | 0.163 | 1.000 | 1.244 | 0.173 | 1.412 | 0.158 | 0.185 |
| 2 | 1.697 | 2.683 | 30 | -0.053 | -0.005 | 0.169 | 0.283 | 0.777 | 0.976 | 1.000 | 1.013 | 0.191 | 0.068 | 0.946 | 0.949 |
| 3 | 2.683 | 3.795 | 32 | -0.053 | 0.034 | 0.159 | 0.542 | 0.588 | 0.839 | 1.000 | 0.817 | 0.192 | 0.952 | 0.341 | 0.329 |
| 4 | 3.795 | 5.434 | 30 | -0.053 | -0.010 | 0.164 | 0.262 | 0.794 | 0.955 | 1.000 | 0.935 | 0.206 | 0.313 | 0.754 | 0.771 |
| 5 | 5.434 | 7.700 | 30 | -0.053 | -0.024 | 0.170 | 0.169 | 0.866 | 0.892 | 1.000 | 1.068 | 0.171 | 0.399 | 0.690 | 0.713 |
| 6 | 7.700 | 10.171 | 32 | -0.053 | 0.048 | 0.152 | 0.663 | 0.507 | 0.718 | 1.000 | 0.915 | 0.223 | 0.379 | 0.705 | 0.695 |

Moran's I correlogram P = 1.00000, Geary's c correlogram P = 0.94806

**ASU Social Sciences Bldg. (Urban)**

| Distance classes | | |  | Moran’s I | | |  |  |  | Geary’s C | | |  |  |  |
| --- | --- | --- | --- | --- | --- | --- | --- | --- | --- | --- | --- | --- | --- | --- | --- |
| # | Min | Max | Pairs | E(I) | I | SD(I) | Z-score | Prob | RandProb | E(c) | c | SD(c) | Z-score | Prob | RandProb |
| 1 | 0 | 1.697 | 30 | -0.053 | 0.108 | 0.163 | 0.989 | 0.323 | 0.538 | 1.000 | 0.933 | 0.178 | 0.373 | 0.709 | 0.718 |
| 2 | 1.697 | 2.683 | 32 | -0.053 | 0.038 | 0.158 | 0.573 | 0.567 | 0.808 | 1.000 | 0.911 | 0.189 | 0.470 | 0.638 | 0.667 |
| 3 | 2.683 | 28.900 | 32 | -0.053 | -0.175 | 0.164 | 0.744 | 0.457 | 0.271 | 1.000 | 1.092 | 0.245 | 0.376 | 0.707 | 0.717 |
| 4 | 28.900 | 31.323 | 32 | -0.053 | -0.375 | 0.143 | 2.258 | 0.024 | 0.024 | 1.000 | 1.712 | 0.300 | 2.372 | 0.018 | 0.014 |
| 5 | 31.323 | 33.721 | 32 | -0.053 | 0.065 | 0.156 | 0.752 | 0.452 | 0.672 | 1.000 | 0.965 | 0.165 | 0.212 | 0.832 | 0.806 |
| 6 | 33.721 | 38.319 | 32 | -0.053 | 0.037 | 0.139 | 0.649 | 0.516 | 0.760 | 1.000 | 0.490 | 0.328 | 1.557 | 0.120 | 0.100 |

Moran's I correlogram P = 0.14371, Geary's c correlogram P = 0.10601
